# Preoperative Carriage of Respiratory Viruses and Acute Respiratory Distress Syndrome After Cardiac Surgery: Protocol for the VIRUS-ATTAC Prospective Cohort Study

**DOI:** 10.2196/103466

**Published:** 2026-09-30

**Authors:** Nicolas Nesseler, Adrien Bouglé, Bertrand Rozec, Nicolas Mongardon, Thomas Kerforne, Chloé Rousseau, Erwan Flécher, Jean-Philippe Verhoye, Mathieu Beaudeau, James Terriss Ross, Marie-Laure Gervais, Claire Fougerou-Leurent, Pierre Tattevin, Vincent Thibault, Célia Pilon, Charlotte Pronier, Alexandre Mansour

**Affiliations:** 1 Department of Anesthesia and Critical Care, Centre Hospitalier Universitaire de Rennes, 11 rue Henri Le Guilloux, Rennes, Brittany, 35000, France, 33 2 99284321; 2 Department of Anesthesia and Critical Care, Pitié-Salpêtrière Hospital, Paris, Île-de-France, France; 3 Department of Anesthesia and Critical Care, Centre Hospitalier Universitaire de Nantes, Nantes, Pays de la Loire, France; 4 Department of Anesthesia and Critical Care, Hôpitaux Universitaires Henri-Mondor, Créteil, Île-de-France, France; 5 Department of Anesthesia and Critical Care, Centre Hospitalier Universitaire de Poitiers, Poitiers, Nouvelle-Aquitaine, France; 6 Department of Pharmacology and Clinical Investigation Center, Centre Hospitalier Universitaire de Rennes, Rennes, Brittany, France; 7 Department of Thoracic and Cardiovascular Surgery, Centre Hospitalier Universitaire de Rennes, Rennes, Brittany, France; 8 Case Western University, University Hospitals of Cleveland, Cleveland, OH, United States; 9 Department of Clinical Research, Centre Hospitalier Universitaire de Rennes, Rennes, Brittany, France; 10 Department of Infectious Diseases, Centre Hospitalier Universitaire de Rennes, Rennes, Brittany, France; 11 Department of Virology, Centre Hospitalier Universitaire de Rennes, Rennes, Brittany, France

**Keywords:** respiratory viruses, influenza, postoperative pulmonary complications, pneumonia, mechanical ventilation, cardiac surgery, acute respiratory distress syndrome, study protocol

## Abstract

**Background:**

Cardiac surgery patients are at particular risk of postoperative acute respiratory distress syndrome (ARDS), with an estimated incidence of 5% to 10%. Cardiopulmonary bypass (CPB), ischemia-reperfusion injury, and blood product transfusions are well-recognized contributing factors. Asymptomatic carriage of respiratory viruses has been hypothesized to prime the lungs and potentiate ARDS development when combined with the pulmonary insults of cardiac surgery. However, no study has directly assessed the relationship between preoperative asymptomatic viral carriage and postoperative ARDS.

**Objective:**

The VIRUS-ATTAC study aims to determine whether asymptomatic carriage of influenza or other respiratory viruses is independently associated with postoperative ARDS following elective cardiac surgery under CPB.

**Methods:**

VIRUS-ATTAC is a prospective, multicenter, low-interventional clinical study conducted across 5 French university hospitals and sponsored by Rennes University Hospital. Adult patients scheduled for elective cardiac surgery under CPB are eligible. A nasopharyngeal flocked swab is collected at anesthesia induction and tested using a multiplex reverse transcription polymerase chain reaction assay detecting 16 respiratory viruses. Results are kept blinded to the clinical team. The primary outcome is ARDS within 7 postoperative days, defined using the Berlin criteria and adjudicated by an independent blinded committee. Secondary outcomes include reintubation, postoperative pulmonary complications, pneumonia, ventilator-free days, intensive care unit–free days, hospital-free days, and 28-day mortality. The primary analysis uses a causal inference framework with a directed acyclic graph for confounder selection and g-computation to estimate the average treatment effect of viral carriage on ARDS risk. A sample size of 1250 patients (including 250 asymptomatic influenza carriers) provides 80% power to detect a 5% absolute risk increase in ARDS (2-sided α=.05).

**Results:**

The VIRUS-ATTAC study received ethics approval from the Comité de Protection des Personnes Sud-Est VI, and recruitment was initiated in February 2021 across 5 French university hospitals. Data collection was completed on March 10, 2023. Of the 1258 patients assessed as eligible, 1204 provided written informed consent and were enrolled. The study database was locked on April 9, 2026. Statistical analysis began after database lock, and the results are expected to be reported in a separate publication in 2027.

**Conclusions:**

VIRUS-ATTAC is the first prospective multicenter study to directly evaluate the association between preoperative asymptomatic respiratory viral carriage and postoperative ARDS in cardiac surgery patients. If confirmed, this association would provide a rationale for evaluating preventive strategies, such as systematic preoperative vaccination or postponement of elective surgery in carriers, in future interventional studies aiming to reduce postoperative respiratory complications.

## Introduction

Acute respiratory distress syndrome (ARDS) is an acute inflammatory lung injury associated with increased pulmonary vascular permeability, increased lung weight, and loss of aerated lung tissue [1]. ARDS-related hospital mortality ranges between 35% and 46% according to disease severity, and survivors are at high risk for cognitive decline, depression, posttraumatic stress disorder, and persistent musculoskeletal weakness [2-4]. Cardiac surgery patients are at particular risk of developing ARDS, with an estimated incidence between 5% and 10% [5-8]. Cardiopulmonary bypass (CPB), lung ischemia-reperfusion, and blood product transfusions are well-recognized factors associated with ARDS development [9-11].

Asymptomatic carriage of respiratory viruses, including influenza virus, has been suspected to be a potential risk factor for pulmonary complications, including ARDS, after cardiac surgery. In a single-center observational cohort study, Groeneveld et al [8] reported that performing elective cardiac surgery during the influenza season was associated with a significantly longer duration of mechanical ventilation and a higher incidence of postoperative ARDS than surgery performed outside the influenza season (odds ratio [OR] 1.85, 95% CI 1.06‐3.23; *P*=.03). Because most influenza infections are asymptomatic—up to 77% in community-based surveillance [12]—the authors hypothesized that asymptomatic carriage of respiratory viruses primes the lungs and, when combined with the pulmonary insults of cardiac surgery, may predispose to ARDS. However, Groeneveld et al [8] did not test individual patients for respiratory viruses, and influenza vaccination coverage in this at-risk Dutch population was high (77.1%), raising the question of whether respiratory viruses other than influenza may also have contributed to the observed effect. A subsequent, larger multicenter cohort by the same group confirmed higher in-hospital mortality among patients operated during the influenza-like illness season, without identifying a specific pulmonary mechanism [13]. This question is clinically relevant because noninfluenza respiratory viruses account for a substantial proportion of influenza-like illness in adults [14], and seasonal variation in cardiac surgery outcomes, including higher winter mortality, has been reported independently of any viral testing [15].

These data are of critical importance. In France, during the 2017‐2018 influenza epidemic season, the vaccination rate of at-risk individuals was only 46%, with an estimated vaccine effectiveness of only 44% in older patients [16]. If an association between carriage of influenza or other respiratory viruses and ARDS is confirmed, an influenza vaccination policy before cardiac surgery, or postponement of elective surgery in the case of a positive test for respiratory virus, might provide a simple and inexpensive strategy to reduce respiratory complications, including ARDS, after cardiac surgery.

Therefore, the primary objective of this study is to estimate the effect of preoperative asymptomatic influenza virus carriage on the risk of postoperative ARDS within 7 days after elective cardiac surgery under CPB. The secondary objectives are to (1) estimate the effect of preoperative asymptomatic carriage of noninfluenza respiratory viruses on the risk of postoperative ARDS within 7 days; (2) estimate the causal effect of preoperative asymptomatic carriage of any respiratory virus (influenza and noninfluenza combined) on the risk of postoperative ARDS within 7 days; and (3) describe whether asymptomatic carriers of respiratory viruses experience other postoperative complications, namely reintubation, postoperative pulmonary complications, pneumonia, ventilator-free days, intensive care unit (ICU)–free days, hospitalization-free days, and mortality at day 28.

## Methods

### Study Design

According to French law, the VIRUS-ATTAC study falls within the scope of a low-interventional clinical study (RIPH2: Recherche Impliquant la Personne Humaine de type 2) that is prospective and multicenter. The study is being conducted in 5 French university centers. The sponsor is the University Hospital of Rennes. Recruitment began in February 2021. The protocol is presented using the SPIRIT (Standard Protocol Items: Recommendations for Interventional Trials) 2013 checklist (Checklist 1). VIRUS-ATTAC is a nonrandomized, low-interventional study (RIPH2); its study-specific procedure is a research nasopharyngeal swab with centralized, masked respiratory virus testing, and SPIRIT items specific to randomized allocation are marked as not applicable. The completed study will be reported following the STROBE (Strengthening the Reporting of Observational Studies in Epidemiology) statement for cohort studies [17].

### Participant Eligibility

Trial site investigators will identify consecutive eligible patients according to the predefined inclusion criteria. Eligible patients will receive written and oral information about the study and will be enrolled after providing written informed consent (Multimedia Appendix 1). The participant eligibility criteria are shown in Textbox 1.

Textbox 1. **Participant eligibility criteria.**

Inclusion criteria

- Patients aged >18 years
- Scheduled for any elective cardiac surgical procedure under cardiopulmonary bypass (eg, coronary artery bypass grafting, valve, aorta, or combined procedures)
- Provision of written informed consent

Exclusion criteria

- Fever or influenza symptoms (headache, myalgias, cough, nasal congestion, rhinorrhea, or sneezing) at preoperative evaluation
- Emergency cardiac surgery
- Heart transplantation
- Left ventricular assist device or total artificial heart implantation
- Adults under legal protection or deprived of liberty

### Study Conduct

For elective cases, patients are typically admitted the day before surgery for preoperative evaluation. Surgery is typically postponed if the patient has a fever or respiratory symptoms; these patients will be excluded. For eligible patients who consent to the study, nasopharyngeal flocked swabs will be collected on the day of surgery, after anesthesia induction and before surgical incision, for viral identification. Results of the assay will be kept blinded to the physician in charge.

In the event of postoperative pneumonia or ARDS, testing for respiratory pathogens, including viruses, will be systematically performed (nasopharyngeal swab or bronchoalveolar lavage) as part of routine care. Results of any postoperative virological assays will be communicated to the physician in charge, and antiviral therapy will be prescribed at the discretion of the attending physician.

### Molecular Assay for Respiratory Virus Detection

Nasopharyngeal flocked swabs are collected in 3 mL of universal transport medium (Copan Diagnostics Inc) and stored at −80 °C until testing. All samples will be sent to Rennes University Hospital for viral detection. A multiplex molecular assay (Allplex Respiratory Panel Assays; Seegene) will be performed according to the manufacturer’s instructions [18]. This assay tests for 16 respiratory viruses: influenza virus types A and B (with influenza A subtyping), adenovirus, coronaviruses HKU1, NL63, 229E and OC43, human metapneumovirus, human rhinovirus or enterovirus, parainfluenza viruses types 1‐4, and respiratory syncytial viruses A and B. The respiratory panel was selected at study inception, before the emergence of SARS-CoV-2. Throughout the study period, SARS-CoV-2 was screened separately as part of routine perioperative care at each center, and a positive preoperative test led to postponement of surgery according to standard institutional practice. SARS-CoV-2 was therefore not included in the study panel.

### Data Collection

Demographic data; data required to calculate the European System for Cardiac Operative Risk Evaluation II (EuroSCORE II) [19], Simplified Acute Physiology Score II [20], and Sequential Organ Failure Assessment scores [21]; CPB duration; perioperative ventilator settings; perioperative blood product infusion; perioperative fluid infusion; vasopressor use and duration; renal replacement therapy use and duration; postoperative mechanical ventilation duration; reintubation (including for surgical revision); postoperative pulmonary complications according to Hulzebos et al [22]; pneumonia defined according to the 2018 French Society of Anesthesia and Intensive Care Medicine (SFAR)–French-speaking Intensive Care Society (SRLF) French guidelines [23]; ICU and hospital length of stay; ICU and hospital mortality; and 28-day mortality will be collected.

### Outcome Measures

#### Primary Outcome Measure

The primary outcome is ARDS within 7 postoperative days, defined according to the Berlin criteria [24]:

- New or worsening respiratory symptoms
- Bilateral opacities on chest radiograph or computed tomography scan—not fully explained by effusions, lobar or lung collapse, or nodules
- Respiratory failure not fully explained by cardiac failure or fluid overload (objective assessment, eg, echocardiography, required to exclude hydrostatic edema)
- Oxygenation impairment, which was evaluated as follows:
  ◦ Mild: 200 mm Hg < arterial partial pressure of oxygen (PaO₂):fraction of inspired oxygen (FiO₂) ratio ≤300 mm Hg with positive end-expiratory pressure (PEEP) or continuous positive airway pressure ≥ 5 cmH₂O
  ◦ Moderate: 100 mm Hg< PaO₂:FiO₂ ratio ≤ 200 mm Hg with PEEP ≥ 5 cmH₂O
  ◦ Severe: PaO₂:FiO₂ ratio ≤ 100 mm Hg with PEEP ≥ 5 cmH₂O

All suspected cases of ARDS will be reviewed by an independent, blinded adjudication committee.

The secondary outcome measures are as follows: reintubation within the first 7 postoperative days; postoperative pulmonary complications of at least grade 2 according to Hulzebos et al [22] within the first 28 postoperative days; pneumonia, defined according to the 2018 SFAR-SRLF French guidelines, within the first 28 postoperative days [23]; ventilator-free days at day 28; ICU-free days at day 28; hospitalization-free days at day 28; and mortality at day 28.

#### Adjudication Committee

The adjudication committee will comprise 3 independent specialists: an intensivist, a pulmonologist, and a radiologist. This committee will be responsible for the blinded assessment and validation of the primary outcome and pneumonia diagnosis. A standardized patient record will be compiled for each case, including all relevant data required for diagnosis. In the event of discordant assessments among committee members, consensus will be sought. If consensus cannot be achieved, a decision will be reached by majority agreement.

### Statistical Analysis

The primary analysis follows a causal inference framework. The target estimand is the average treatment effect of asymptomatic influenza virus carriage on the risk of ARDS within 7 postoperative days. The causal model is specified through a directed acyclic graph (DAG) reflecting the assumed causal structure relating preoperative viral carriage, postoperative ARDS, and potential confounders (Figure 1). The primary analysis will use a multivariable logistic regression model, fitted with ARDS at day 7 as the outcome; asymptomatic influenza carriage as the exposure; and age, chronic obstructive pulmonary disease, immunosuppression, and influenza vaccination status as confounders (identified from the DAG). G-computation [25] will be used to compute the predicted probability of ARDS for each patient under both counterfactual scenarios (carrier vs noncarrier). Center will be modeled as a random intercept to account for between-center variation in case mix and clinical practice, reflecting a design effect rather than a confounder. The same analytical approach will be applied for the secondary objectives concerning noninfluenza viral carriage and any-virus carriage. Covariate selection followed the back-door criterion applied to the DAG. Perioperative variables (surgical procedure type, EuroSCORE II, CPB duration, intraoperative transfusion, and baseline left ventricular ejection fraction) were excluded because they lie downstream of the outcome or are not common causes of both exposure and ARDS; conditioning on them would risk overadjustment or collider bias. Sex, smoking status, and the Charlson comorbidity index were excluded because we found no evidence that they are common causes of both carriage and ARDS.

Given the expected low mortality within the 7-day primary end point window in this elective cardiac surgery population, death before day 7 is unlikely to substantially bias the primary analysis. Patients who die before day 7 without a prior ARDS diagnosis will be classified as not having developed ARDS. A sensitivity analysis using a Fine and Gray subdistribution hazard model [26] treating death as a competing event will be conducted to assess robustness.

The feasibility of the influenza-specific primary analysis depends on the observed prevalence of asymptomatic influenza carriage, which may vary substantially due to seasonal variation and the potential impact of the COVID-19 pandemic on respiratory virus circulation during the recruitment period (2021‐2023) [27,28]. The following prespecified decision rule will be applied based on exposure prevalence, without knowledge of the outcome distribution:

- If ≥100 asymptomatic influenza carriers are observed: the primary analysis will proceed as specified.
- If <100 asymptomatic influenza carriers but ≥100 carriers of any respiratory virus are observed: the primary analysis will be redirected to asymptomatic carriage of any respiratory virus as the exposure, with the same analytical framework. The influenza-specific analysis will be reported as exploratory.
- If <100 carriers of any respiratory virus are observed: only descriptive analyses will be performed (crude proportions with exact 95% CIs and baseline comparison using standardized mean differences).

Secondary outcomes will be reported descriptively by exposure group (carriers vs noncarriers). No multivariable modeling or hypothesis testing will be performed for secondary outcomes, as these analyses are exploratory and not supported by a formal sample size calculation. For binary outcomes (reintubation, postoperative pulmonary complications, pneumonia, and 28-day mortality), crude proportions with 95% CIs will be provided for each group, along with unadjusted risk differences with 95% CIs. For free-day outcomes (ventilator-free days, ICU-free days, and hospital-free days at day 28), medians with IQRs will be reported by group; patients who die before day 28 will be assigned zero free days.

Missing data on adjustment covariates will be handled using multiple imputation by chained equations [29]. The imputation model will include the outcome, exposure, all adjustment covariates, and auxiliary variables predictive of missingness. Rubin rules will be used to pool the estimates. Missing data are assumed to be missing at random conditional on the observed variables. The proportion and pattern of missing data will be reported for all variables.

Continuous variables will be described as means with SDs or medians with IQRs, as appropriate. Categorical variables will be described as counts and percentages. Baseline characteristics will be compared between patients with and without viral carriage using standardized mean differences [30].

**Figure 1.**
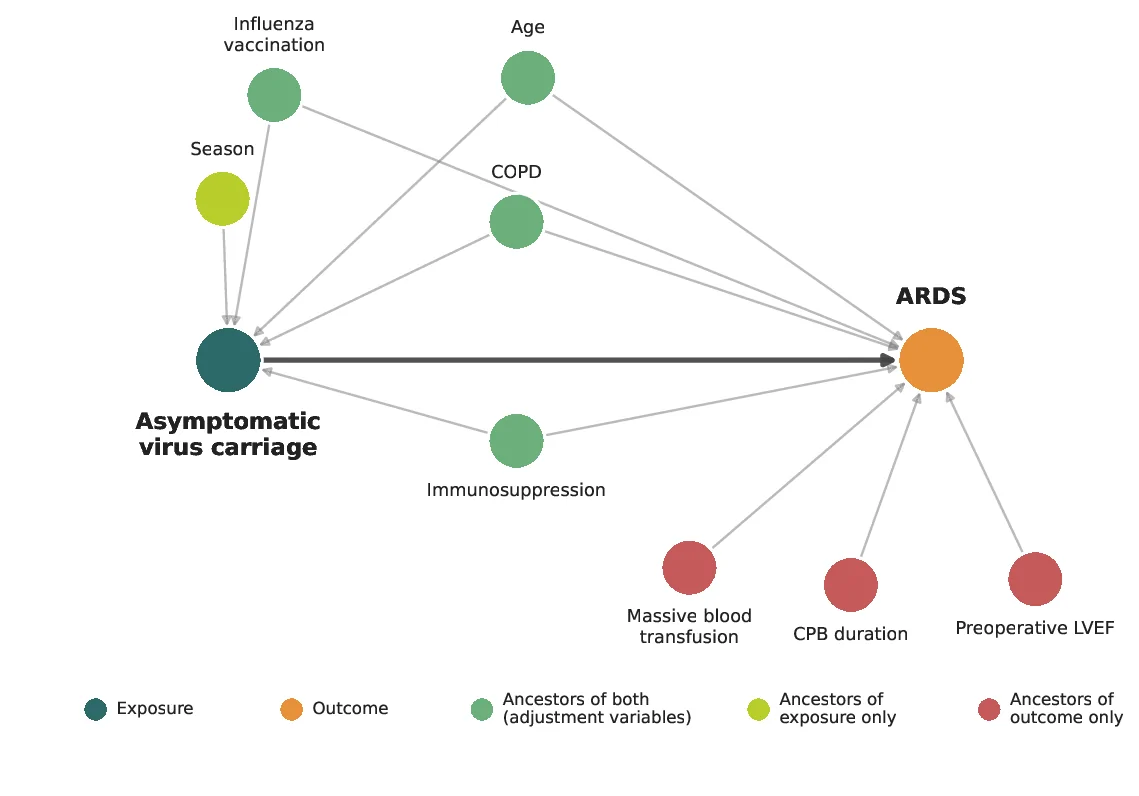
Directed acyclic graph (DAG). The causal model is specified through a DAG reflecting the assumed causal structure relating preoperative viral carriage, postoperative acute respiratory distress syndrome (ARDS), and potential confounders. On the basis of the DAG, the minimal sufficient adjustment set for the relationship between viral carriage and postoperative ARDS consists of age, chronic obstructive pulmonary disease (COPD), immunosuppression, and influenza vaccination status. CPB: cardiopulmonary bypass; LVEF: left ventricular ejection fraction.

### Sample Size

A sample size of 1250 patients, including 250 asymptomatic influenza carriers, provides 80% power to detect an absolute risk increase in ARDS of 5% in asymptomatic carriers, using a significance level of .05 (2-sided). This assumes a 20% prevalence of asymptomatic influenza carriage in the general population and a 5% prevalence of ARDS after cardiac surgery [8,31]. Because recruitment took place between 2021 and 2023, a period during which nonpharmaceutical interventions against COVID-19 markedly reduced the circulation of influenza and other respiratory viruses [27], the observed prevalence of asymptomatic carriage may be lower than this assumption. The prespecified decision rule described in the Statistical Analysis section ensures that the study remains informative if carriage is lower than expected by redirecting the primary analysis to any respiratory virus carriage or, if necessary, to descriptive analyses.

### Ethical Considerations

#### Ethics Approval and Confidentiality of Data

The VIRUS-ATTAC study has been approved for all centers by a central ethics committee (Comité de Protection des Personnes Sud-Est VI). The study is registered on ClinicalTrials.gov (NCT04562207) in accordance with the World Health Organization trial registration dataset recommendations.

Masking of the virological results to the treating team raises no additional risk to participants and is ethically justified. The multiplex assay is performed on batched samples centralized at Rennes University Hospital and is not available in real time; therefore, the results could not guide perioperative management even if they were unmasked. Clinical equipoise applies because there is currently no evidence that detecting asymptomatic respiratory virus carriage should modify the decision to operate or the perioperative management of otherwise eligible patients. Symptomatic patients are excluded at screening and managed according to standard care, and any respiratory pathogen identified clinically during the postoperative course is tested and treated independently of the study. SARS-CoV-2 is screened separately as part of routine perioperative care. Findings relevant to a participant’s care are communicated according to standard clinical practice.

In accordance with Good Clinical Practice, the French Public Health Code, and European Regulation No. 2016/679 of May 25, 2018, relating to data protection, the sponsor is responsible for obtaining the agreement of all parties involved in the research to guarantee direct access to all research locations, source data, source documents, and reports for quality control and audit purposes. Persons with direct access will take all necessary precautions to ensure the confidentiality of information relating to the products, trials, persons involved, and results obtained, as these persons are subject to professional secrecy (under the conditions defined by articles 226‐13 and 226‐14 of the French Penal Code).

In accordance with the General Data Protection Regulation (EU) 2016/679, data collected from participants and transmitted to the sponsor will be pseudonymized. Under no circumstances will the names or addresses of the participants appear in clear text. Only the first letter of each participant’s last and first name will be recorded, along with a trial-specific coded number indicating the order of participant inclusion.

#### Data Management

All collected data will be recorded in a secure web-based electronic case report form (eCRF) by study or clinical personnel under investigator supervision. The study database will be established from the eCRF, and data collection will be monitored by trained clinical research assistants. All original records (including consent forms and relevant correspondences) will be archived at trial sites for 15 years. The clean trial database file will be pseudonymized and maintained for 15 years.

The final study dataset will be accessible to the principal investigators and designated members of the research team at Rennes University Hospital. Deidentified data may be made available upon reasonable request following institutional and ethical guidelines. There are no contractual agreements restricting investigators’ access to the data.

#### Patient Withdrawal

Participants may withdraw consent at any time without providing a reason. Those withdrawing will continue to receive standard clinical care in each participating center. To conduct analyses with the minimum of missing data, investigators may ask if the participant is willing to continue follow-up assessments and allow use of previously collected data. Whenever possible, the participant will be asked for permission to obtain data for the primary outcome measure. All included patients will be reported, and all data for which consent has been provided will be used in the analyses.

#### Patient and Public Involvement

Patients and the public were not involved in any phase of this study.

### Dissemination Plan

The results of VIRUS-ATTAC will be disseminated through publications in peer-reviewed international journals and presentations at congresses in anesthesiology, intensive care, and cardiothoracic surgery and will be reported in accordance with the STROBE statement for cohort studies [17]. Summary results will be posted on ClinicalTrials.gov (NCT04562207) and communicated to the participating centers.

## Results

The VIRUS-ATTAC study was registered on ClinicalTrials.gov (NCT04562207) in 2020 and received ethics approval from the Comité de Protection des Personnes Sud-Est VI prior to study initiation. The study was funded between 2019 and 2020. Recruitment began on February 9, 2021, across 5 French university hospitals and was completed on March 10, 2023. Of the 1258 patients assessed as eligible, 1204 (96%) provided written informed consent and were enrolled; the remaining 54 (4%) eligible patients were not enrolled for reasons that were not systematically documented at the time. No study data were collected for patients who were not enrolled. The study database was locked on April 9, 2026. Statistical analysis began after database lock, and the results are expected to be reported in a separate publication in 2027.

## Discussion

### Principal Findings

We hypothesize that preoperative asymptomatic carriage of influenza or other seasonal respiratory viruses is independently associated with an increased risk of postoperative ARDS within 7 days of elective cardiac surgery under CPB. VIRUS-ATTAC is designed to test this hypothesis directly; this section outlines the anticipated contribution of the study rather than its results, which are not yet available. To our knowledge, VIRUS-ATTAC is the first investigator-initiated, prospective, multicenter cohort study to evaluate directly the impact of preoperative asymptomatic carriage of influenza and other respiratory viruses on the risk of postoperative ARDS following elective cardiac surgery under CPB.

### Comparison With Prior Work

To date, the most relevant evidence on the association between respiratory viral exposure and postoperative ARDS after cardiac surgery comes from a single-center observational study by Groeneveld et al [8], who found that performing elective cardiac surgery during influenza season was associated with increased ARDS incidence (OR 1.85, 95% CI 1.06‐3.23). However, that study relied on seasonal exposure as a surrogate for viral carriage without virological testing, could not distinguish between influenza and other circulating respiratory viruses, and did not adjust for potential confounders using a causal framework. Moreover, the high influenza vaccination rate in that population (77%) may have attenuated the true effect of viral carriage. VIRUS-ATTAC addresses these limitations by directly testing each patient with multiplex RT-PCR, covering 16 respiratory viruses, blinding the clinical team, and applying a formal causal inference approach to the analysis.

### Strengths and Limitations

VIRUS-ATTAC has several methodological strengths: a prospective multicenter design, systematic virological testing of all enrolled patients irrespective of symptoms, masking of virological results to the clinical team, independent blinded outcome adjudication, centralized virological analysis, and a prespecified causal inference framework. Virological results are kept blinded to the clinical team throughout the perioperative period, thereby preventing differential management and outcome ascertainment bias. The primary outcome (ARDS) and pneumonia diagnosis are adjudicated by an independent blinded committee comprising an intensivist, a pulmonologist, and a radiologist, following the PROBE (Prospective Randomized Open Blinded End-point) methodology. Centralization of all virological analyses at Rennes University Hospital ensures standardized and harmonized processing across all study sites. The primary analysis uses a causal inference framework, with DAG-based confounder selection and g-computation, to estimate the average treatment effect of viral carriage on postoperative ARDS, which is more robust to confounding than standard multivariable adjustment. This study has several limitations. First, asymptomatic viral carriage is only investigated in elective cardiac surgery patients, which may not reflect the full cardiac surgery population, including emergency and urgent cases. Second, the prevalence of viral carriage is highly dependent on the respiratory virus season and may be affected by year-to-year variability in circulating viruses; in particular, the COVID-19 pandemic substantially disrupted respiratory virus circulation during the recruitment period (2021‐2023), potentially affecting the prevalence of influenza and other respiratory viruses and the representativeness of the sample. These factors may lead to uneven distribution of positive cases across recruitment periods and centers, which is addressed in the prespecified analytical decision rule. In addition, because the panel was defined before the COVID-19 pandemic, SARS-CoV-2 was not among the assayed viruses; it was screened separately as part of routine perioperative care, and patients testing positive had their surgery postponed and thus did not enter the operated cohort [32].

### Future Directions

If VIRUS-ATTAC confirms an independent association between asymptomatic respiratory virus carriage and postoperative ARDS, several lines of research would follow. Interventional trials could test preventive strategies such as systematic preoperative virological screening with postponement of elective surgery in confirmed carriers or systematic preoperative influenza vaccination [33]. The question could be extended to urgent and emergency cardiac surgery and to other major surgical procedures, in which preoperative screening and postponement are not feasible. Mechanistic substudies could clarify how subclinical viral carriage interacts with the pulmonary insults of CPB to precipitate lung injury.

### Conclusions

If an independent association between preoperative asymptomatic respiratory virus carriage and postoperative ARDS is confirmed, this would provide a rationale for evaluating preventive strategies, such as systematic preoperative influenza vaccination or postponement of elective surgery in virologically confirmed carriers, in future interventional trials aiming to reduce postoperative respiratory complications after cardiac surgery.

## Supplementary material

10.2196/103466 Multimedia Appendix 1 Patient consent.

10.2196/103466 Checklist 1 SPIRIT checklist.

## Abbreviations

ARDS: acute respiratory distress syndrome
CPB: cardiopulmonary bypass
DAG: directed acyclic graph
eCRF: electronic case report form
EuroSCORE II: European System for Cardiac Operative Risk Evaluation II
FiO₂: fraction of inspired oxygen
ICU: intensive care unit
OR: odds ratio
PaO₂: arterial partial pressure of oxygen
PEEP: positive end-expiratory pressure
PROBE: Prospective Randomized Open Blinded End-point
SFAR: French Society of Anesthesia and Intensive Care Medicine
SPIRIT: Standard Protocol Items: Recommendations for Interventional Trials
SRLF: French-speaking Intensive Care Society
STROBE: Strengthening the Reporting of Observational Studies in Epidemiology
