# Supplementary material for: Preoperative Carriage of Respiratory Viruses and Acute Respiratory Distress Syndrome After Cardiac Surgery: Protocol for the VIRUS-ATTAC Prospective Cohort Study
Source: JMIR Res Protoc. 2026 Sep 30;15:e103466. doi: 10.2196/103466 (PMC13626548; doi:10.2196/103466)
Supplement: Supplementary file 1 [file resprot-v15-e103466-s002.pdf]

Multimedia Appendix: SPIRIT 2013 Checklist

VIRUS-ATTAC — Pre-operative Carriage of Respiratory Viruses and Acute Respiratory Distress Syndrome After Cardiac Surgery: Protocol of a Prospective Cohort Study.

VIRUS-ATTAC is a prospective multicentre cohort study classified as low-interventional under French law (RIPH2). Its study-specific procedure is a single research nasopharyngeal swab with centralised, masked respiratory-virus testing; the study is non-randomised and comprises a single cohort. The SPIRIT 2013 checklist was developed for randomised clinical trial protocols and is provided here as recommended by the editor. Items specific to randomised allocation (sequence generation, allocation concealment, implementation, and emergency unblinding) are marked "N/A — non-randomised study (single cohort)." Locations refer to manuscript sections; page numbers will be inserted at typesetting.

Item No	Checklist item (SPIRIT 2013)	Location in manuscript / status
**Administrative information**		
1	Descriptive title identifying the study design, population, and, if applicable, registration	Title page
2a	Trial identifier and registry name; if not yet registered, name of intended registry	Methods, Ethical Considerations (ClinicalTrials.gov, NCT04562207)
2b	All items from the WHO Trial Registration Data Set	ClinicalTrials.gov record (NCT04562207)
3	Date and version identifier	Title page / ClinicalTrials.gov record (NCT04562207)
4	Sources and types of financial, material, and other support	Funding
5a	Names, affiliations, and roles of protocol contributors	Title page; Authors' Contributions
5b	Name and contact information for the trial sponsor	Methods, Ethical Considerations (sponsor: CHU de Rennes)
5c	Role of study sponsor and funders in design, conduct, analysis, reporting, and decision to publish	Funding (sponsor and funders had no role in analysis or the decision to publish)
5d	Composition, roles, and responsibilities of coordinating centre, steering and other committees	Methods, Adjudication Committee (independent blinded committee); sponsor CHU de Rennes
**Introduction**		
6a	Background and rationale, including a summary of relevant studies	Introduction
6b	Explanation for choice of comparators	Exposure groups (respiratory-virus carriers vs non-carriers) defined by the study procedure; no therapeutic comparator (Methods)
7	Specific objectives or hypotheses	Introduction (objectives); Methods
8	Description of study design including type and framework	Methods, Study Design (prospective multicentre cohort; low-interventional study, RIPH2)
**Methods: Participants, interventions, and outcomes**		
9	Description of study settings and list of sites	Methods, Study Design (five French university hospitals)
10	Inclusion and exclusion criteria for participants	Methods, Participant Eligibility
11a	Interventions for each group with detail to allow replication	Study-specific procedure: research

Item No	Checklist item (SPIRIT 2013)	Location in manuscript / status
		nasopharyngeal swab collected on the day of surgery (after anaesthesia induction, before incision), with centralised, masked Allplex 16-virus testing (Methods, Study Conduct and Molecular Assay). Single cohort; no therapeutic intervention or comparator group
11b	Criteria for discontinuing or modifying allocated interventions	N/A — single, one-time study procedure
11c	Strategies to improve adherence to intervention protocols	Procedure performed intraoperatively by trained site teams (Methods, Study Conduct)
11d	Relevant concomitant care permitted or prohibited	Methods, Study Conduct (standard perioperative care)
12	Primary, secondary, and other outcomes with metric, method of aggregation, and time point	Methods, Outcome Measures
13	Time schedule of enrolment, interventions, assessments, and visits	Methods, Study Conduct and Data Collection (perioperative to day 28); Results
14	Estimated number of participants needed and how it was determined	Methods, Sample Size
15	Strategies for achieving adequate participant enrolment to reach target sample size	Methods, Participant Eligibility and Study Conduct (consecutive eligible patients); Results
**Methods: Assignment of interventions (for controlled trials)**		
16a	Sequence generation	N/A — non-randomised study (single cohort)
16b	Allocation concealment mechanism	N/A — non-randomised study (single cohort)
16c	Implementation of allocation sequence	N/A — non-randomised study (single cohort)
17a	Who will be blinded and how	No randomised allocation to blind; viral assay results masked to the treating physician (Methods, Study Conduct and Molecular Assay) and outcome adjudication performed blinded (Methods, Adjudication Committee)
17b	Circumstances and procedure for emergency unblinding	N/A — non-randomised study (single cohort)
**Methods: Data collection, management, and analysis**		
18a	Plans for assessment and collection of outcome, baseline, and other data	Methods, Data Collection; Molecular Assay; Outcome Measures
18b	Plans to promote participant retention and complete follow-up	Methods, Patient Withdrawal
19	Plans for data entry, coding, security, and storage	Methods, Data Management (secure web-based eCRF)
20a	Statistical methods for analysing primary and secondary	Methods, Statistical Analysis

Item No	Checklist item (SPIRIT 2013)	Location in manuscript / status
	outcomes	
20b	Methods for any additional analyses (e.g., subgroup, sensitivity)	Methods, Statistical Analysis (Fine-Gray competing-risk sensitivity analysis; pre-specified decision rule)
20c	Definition of analysis population and statistical methods to handle missing data	Methods, Statistical Analysis (multiple imputation by chained equations; Rubin's rules)
**Methods: Monitoring**		
21a	Composition of data monitoring committee and its role	Methods, Data Management (data monitoring by sponsor); no formal DMC, consistent with the low-risk (RIPH2) design
21b	Description of any interim analyses and stopping guidelines	N/A — no interim analysis planned
22	Plans for collecting, assessing, reporting, and managing solicited and spontaneous adverse events	The only study procedure is a single nasopharyngeal swab (minimal risk); adverse events related to the procedure are reported to the sponsor per RIPH2 vigilance requirements. Clinical events are managed as routine perioperative care (Methods, Study Conduct and Ethical Considerations)
23	Frequency and procedures for auditing trial conduct	Methods, Data Management (sponsor monitoring of data collection)
**Ethics and dissemination**		
24	Plans for seeking research ethics committee / IRB approval	Methods, Ethical Considerations (Comité de Protection des Personnes Sud-Est VI)
25	Plans for communicating important protocol modifications to relevant parties	Managed by the sponsor (CHU de Rennes) per standard procedures; registry updated
26a	Who will obtain informed consent and how	Methods, Participant Eligibility (written informed consent); Ethical Considerations
26b	Consent provisions for collection and use of data and biological specimens in ancillary studies	N/A — no ancillary studies; specimens used only for the protocol assay
27	How confidentiality of personal information will be maintained	Methods, Ethical Considerations and Data Management (pseudonymisation; GDPR (EU) 2016/679)
28	Financial and other competing interests for principal investigators	Conflicts of Interest
29	Statement of who will have access to the final dataset	Methods, Data Management (principal investigators and designated team; de-identified data on reasonable request)
30	Provisions for ancillary and post-trial care	Participants continue standard

Item No	Checklist item (SPIRIT 2013)	Location in manuscript / status
		clinical care; the single study procedure entails no post-trial care obligation (Methods, Patient Withdrawal and Ethical Considerations)
31a	Plans for communicating results to participants, professionals, public, and other groups	Discussion, Dissemination Plan (peer-reviewed journals, congresses, ClinicalTrials.gov results posting)
31b	Authorship eligibility guidelines and any use of professional writers	Authors' Contributions (ICMJE criteria; no professional writers)
31c	Plans for granting public access to the full protocol, dataset, and code	Discussion, Dissemination Plan (de-identified data on reasonable request, per sponsor policy and GDPR)
**Appendices**		
32	Model consent form and other related documentation given to participants	Available from the sponsor (CHU de Rennes) on request; not included
33	Plans for collection, laboratory evaluation, and storage of biological specimens	Methods, Molecular Assay and Data Collection (nasopharyngeal swabs in UTM, stored at -80°C, centralised at CHU de Rennes)

Source of the checklist (according to PubMed): Chan AW, Tetzlaff JM, Altman DG, et al. SPIRIT 2013 statement: defining standard protocol items for clinical trials. Ann Intern Med. 2013;158(3):200-207. doi:10.7326/0003-4819-158-3-201302050-00583.
