# Supplementary material for: Preoperative Carriage of Respiratory Viruses and Acute Respiratory Distress Syndrome After Cardiac Surgery: Protocol for the VIRUS-ATTAC Prospective Cohort Study
Source: JMIR Res Protoc. 2026 Sep 30;15:e103466. doi: 10.2196/103466 (PMC13626548; doi:10.2196/103466)
Supplement: Supplementary file 2 [file resprot-v15-e103466-s001.docx]

**LETTRE D’INFORMATION**

**POUR LA PARTICIPATION A UNE RECHERCHE CLINIQUE**

**VIRUS-ATTAC**

**Portage pre-operatoire de VIRUS respirAToires, et syndrome de détresse respiraToire Aiguë après chirurgie Cardiaque**

| **Catégorie de la recherche** : Recherche impliquant la personne humaine à risques et contraintes minimes | |
| --- | --- |
| Réf. CHU de Rennes : 35RC19_8869_VIRUS-ATTAC | N° ID-RCB : 2020-A00345-34 |
| **Promoteur :** CHU de Rennes  **Contact :** Direction de la Recherche et de l’Innovation  Hôpital de Pontchaillou  2 rue Henri le Guilloux  35 033 Rennes Cedex 9  Tél. : 02.99.28.25.55  **Délégué à la Protection des Données :** [dpo@chu-rennes.fr](mailto:dpo@chu-rennes.fr) | **Investigateur Coordonnateur :**  **Dr Nicolas NESSELER**  Service de Anesthésie-réanimation 3 – Réanimation CTCV  Centre cardio-pulmonaire, Hôpital Pontchaillou  2 avenue Henri Le Guilloux  35033 RENNES cedex 9  Tél. : 02.99.28.91.53 |

Madame, Monsieur,

Vous avez été invité(e) à participer à l’étude clinique intitulée VIRUS-ATTAC. Le CHU de RENNES est le promoteur de cette étude, il en est responsable et en assure l’organisation.

Avant de décider de participer à cette étude, il est important pour vous d’en comprendre l’objectif ainsi que ses implications. Cette lettre d’information est destinée à vous aider à prendre une décision concernant votre participation à l’essai qui vous est proposé. Prenez le temps de lire attentivement les informations suivantes, et d’en discuter avec vos proches. Si toutefois certains points manquent de clarté après la lecture de cette lettre d’information et l’information orale qui vous a été donnée sur le protocole, ou si vous avez besoin d’informations complémentaires, n’hésitez pas à en parler avec votre médecin investigateur. Vous pouvez prendre tout le temps nécessaire avant de décider si vous souhaitez participer entre la remise de cette lettre et le jour de votre chirurgie.

Si vous décidez de participer à cette recherche, vous devrez signer un formulaire de consentement. Cette signature confirmera que vous êtes d’accord pour participer à cette étude.

Si vous faites l’objet d’une curatelle, tutelle ou sauvegarde de justice, vous ne pouvez pas participer à l’étude, aussi nous vous demandons d’en informer sans tarder votre médecin investigateur.

1- **OBJECTIF DE L’ETUDE**

Le syndrome de détresse respiratoire de l’adulte (SDRA) est une complication respiratoire grave observée après une chirurgie cardiaque chez 5% des patients. Récemment une étude néerlandaise a mis en évidence une possible augmentation de ce type de complications chez les patients bénéficiant d’une chirurgie cardiaque en période d’épidémie grippale. Cependant dans ce travail, les patients n’étaient pas testés pour la grippe et la responsabilité des virus respiratoires non-grippaux ne peut être exclue. Confirmer une association entre virus grippal et/ou virus respiratoires non grippaux et complications respiratoires postopératoires pourraient constituer une avancée très importante. Celle-ci permettrait la mise en place de stratégies simples et peu coûteuses permettant la réduction des complications respiratoires.

L’objet de ce projet de recherche est donc de dépister, en ayant recours aux plus récentes techniques de diagnostic moléculaire viral, le portage asymptomatique préopératoire de virus respiratoires grippaux et non-grippaux (hors virus du COVID19) et de déterminer s’il existe une association entre virus respiratoires grippaux et/ou non grippaux et SDRA ou complications respiratoires après chirurgie cardiaque.

Nous prévoyons une période de recrutement de 30 mois avec une durée de suivi des patients de 28 jours. Le nombre de participants prévu est de 1250 patients.

Si vous présentez de la fièvre ou des symptômes grippaux (céphalées, myalgies, toux, congestion nasale, rhinorrhée), parlez-en à votre médecin investigateur, vous pourriez ne pas pouvoir participer.

2- **DEROULEMENT DE L’ETUDE**

Votre médecin investigateur vous propose de participer à cette étude au cours d’une consultation préopératoire ou lors de votre admission la veille de votre chirurgie. Après information orale il vous a remis cette lettre d’information. Après avoir pris le temps de la réflexion et posé vos éventuelles questions au médecin investigateur, si vous consentez à participer, vous signerez le formulaire de consentement joint à cette lettre et vous serez inclus dans l’étude avant votre chirurgie cardiaque. Avant la chirurgie, la veille ou au bloc opératoire, un écouvillon nasopharyngé sera collecté. Cet examen indolore appartient à la famille des tests de diagnostic rapide. Il consiste à insérer un écouvillon (petite brosse semblable à un coton-tige) dans une narine pour réaliser un prélèvement non invasif le long de la paroi. Le prélèvement peut être réalisé la veille ou le jour de la chirurgie, vous pourriez donc être déjà sous anesthésie ou inconscient au moment du prélèvement. L’écouvillon est ensuite mis dans une solution de stockage et transféré au laboratoire de virologie du CHU de Rennes pour analyse.

L’analyse virologique sera réalisée en différé de votre inclusion pour des raisons de faisabilité et compte tenu du fait que cette analyse n’a pas de lien direct avec votre prise en charge. Elle permettra de détecter la présence de 16 types de virus respiratoires, dont la grippe. Les résultats ne seront donc pas communiqués au médecin investigateur durant votre participation.

Afin de suivre les potentielles complications que vous auriez eu, et notamment respiratoires, le médecin investigateur recueillera lors de votre consultation de suivi après 28 jours votre état de santé depuis la chirurgie.

Aucune autre procédure ne sera réalisée. Votre prise en charge n’est pas modifiée. La participation à une autre étude est possible sous conditions, mais elle ne doit pas impacter votre participation à VIRUS-ATTAC. Si vous participez déjà à une autre étude clinique, ou si vous souhaitez durant votre participation à VIRUS-ATTAC participer à une autre étude clinique, parlez-en à votre médecin investigateur.

A l’issue de l’étude, et à votre demande, vous pourrez être informé(e) des résultats globaux de la recherche par votre médecin investigateur.

3- **RISQUES POTENTIELS DE L’ETUDE**

La participation au protocole VIRUS-ATTAC vous expose uniquement aux risques liés au prélèvement nasopharyngé effectué, non invasif. Le seul effet indésirable attendu est un inconfort relatif pour le patient lors de l’examen. Cependant, la majorité des écouvillonnages seront réalisés sous anesthésie générale après induction anesthésique et avant l’incision chirurgicale. Il n’y a pas de risque psychologique ou socio-économique.

Cet examen est pratiqué de façon courante par les médecins et infirmières du service après chirurgie en cas de suspicion d’infection respiratoire.

4- **BENEFICES POTENTIELS DE L’ETUDE**

Aucun bénéfice personnel immédiat pour les patients inclus de l’étude n’est attendu dans le cadre de cette recherche.

Les résultats de cette recherche représentent un bénéfice collectif au sens d’une avancée des connaissances dans un intérêt collectif. Le bénéfice collectif sera une meilleure prévention et appréhension des complications post-opératoires respiratoires suite à des chirurgies cardiaques sous circulation extracorporelle.

5- **PARTICIPATION VOLONTAIRE**

Votre participation à cette étude est entièrement volontaire et n’engendre aucun surcoût à votre charge.

Vous êtes libre de refuser d’y participer ainsi que de mettre un terme à votre participation à l’étude à n’importe quel moment, sans encourir aucun préjudice de ce fait et sans que cela n’entraîne de conséquences sur la qualité des soins qui vous seront prodigués.

Dans ce cas, vous devez informer le médecin de votre décision.

Dans le cas où vous retirez votre consentement, nous effectuerons un traitement informatique de vos données personnelles déjà recueillies, sauf opposition écrite de votre part. En revanche, nous considérerons que votre refus s’applique sur la réutilisation ultérieure ou le partage des données.

Vos échantillons seront conditionnés et conservés pendant 5 ans, après la dernière publication des résultats, au sein du laboratoire de virologie du CHU de Rennes. A l’issue des 5 ans suivant la dernière publication des résultats de la recherche, les échantillons seront détruits. Dans le cadre d’un potentiel bénéfice collectif ultérieur de votre participation à VIRUS-ATTAC, nous souhaiterions avoir votre autorisation de réutiliser vos échantillons à des fins de recherche ultérieures.

Si vous l’autorisez, votre médecin traitant sera informé de votre participation à cette étude.

Durant l’étude, vous serez averti(e) par votre médecin investigateur des faits nouveaux pouvant affecter votre volonté de participer à l’étude.

Les Autorités de Santé, votre médecin investigateur ou le promoteur peuvent décider de mettre un terme à votre participation à l’étude à n’importe quel moment sans votre consentement préalable. Si cela devait se produire, vous en serez averti(e) et les raisons vous seraient expliquées*.*

6- **CONFIDENTIALITE ET UTILISATION DES DONNEES PERSONNELLES**

Dans le cadre de la recherche à laquelle le CHU de Rennes et votre médecin vous proposent de participer, vos données personnelles seront transmises, traitées et analysées au regard des objectifs qui vous ont été présentés.

Ces données pourront également être transmises aux autorités de santé françaises.

A cette fin, les données médicales recueillies seront transmises au Promoteur de la recherche ou à ses partenaires (personnes ou sociétés agissant pour son compte) en France.

Ces données pourront également être transmises aux autorités de santé françaises, à leur demande en cas d’inspection.

Par souci de confidentialité, ces données seront identifiées par un numéro de code et/ou vos initiales.

Cette étude entre dans le cadre de la « Méthodologie de Référence MR-001 » établi par la Commission Nationale Informatique et Liberté (CNIL). Le CHU de Rennes, en tant que promoteur de l’étude, a signé un engagement de conformité à cette « Méthodologie de Référence » qui garantit que le traitement des données personnelles suit bien les exigences de la CNIL.

Le personnel impliqué dans l’étude est soumis au secret professionnel, tout comme votre médecin traitant.

Conformément aux dispositions de loi relative à l’informatique, aux fichiers et aux libertés (loi du 6 janvier 1978 modifiée) et du Règlement Européen 2016/679 du 27 avril 2016, vous disposez d’un droit d’accès, de portabilité, de rectification, d’effacement et de limitation de vos données personnelles. Toutefois, si l’effacement de vos données est susceptible de rendre impossible ou de compromettre la réalisation des objectifs de la recherche, les données collectées antérieurement à votre opposition seront conservées, conformément au Règlement Général de Protection des Données (art. 17.3 c et 17.3 d) de l’Union Européenne n°2016/679.

Le CHU de RENNES est *le responsable de traitement* au sens du Règlement Européen 2016/679, puisqu’il détermine les finalités et les moyens du traitement des données dans le cadre de cette étude. L’article 9 de ce règlement lui permet de traiter des catégories particulières de données, incluant des données de santé.

Vous disposez également d’un droit d’opposition à la transmission des données couvertes par le secret professionnel susceptibles d’être utilisées dans le cadre de cette recherche et d’être traitées.

Les données vous concernant seront conservées pendant une durée de 15 ans à compter de la fin de l’étude.

Vous pouvez également accéder directement ou par l’intermédiaire d’un médecin de votre choix à l’ensemble de vos données médicales en application des dispositions de l’article L 1111-7 du Code de la Santé Publique.

Ces droits s’exercent auprès du médecin qui vous suit dans le cadre de la recherche et qui connaît votre identité ou du Délégué à la Protection des Données du CHU de Rennes (dpo@chu-rennes.fr).

Pour toute réclamation relative au traitement de vos données de santé, vous pouvez saisir la CNIL (<https://www.cnil.fr/fr/webform/adresser-une-plainte>).

**Partage des données personnelles anonymisées**

Dans le respect de la réglementation en vigueur et dans un esprit de partage du bien commun que représentent les données d’une recherche, une mise à disposition de vos données personnelles anonymisées est envisagée à des journaux scientifiques qui en feraient la demande (à destination de rapporteurs et experts dans le domaine étudié).

**Utilisation des données personnelles à des fins de recherches ultérieures**

Si vous ne vous y opposez pas, vos données personnelles issues du projet VIRUS-ATTAC pourront être utilisées à des fins de recherches ultérieures. Pour chaque nouvelle recherche s’appuyant sur ces données, l’intérêt scientifique et le respect de la réglementation en vigueur seront vérifiés. Chaque recherche autorisée fera l’objet d’une information collective sur le site internet du CHU de Rennes (<https://www.chu-rennes.fr>).

7- **ASPECTS LEGAUX**

Un contrat d’**assurance** N° 0100661314039 200001 a été souscrit par le CHU de Rennes, auprès de la compagnie d’assurance HDI Global SE, pour couvrir les risques liés à cette recherche. Cette assurance couvre non seulement la responsabilité du CHU de Rennes en tant que promoteur d’une recherche interventionnelle impliquant la personne humaine mais également celle de tout autre intervenant, en accord avec l’article L 1121-10 du Code de la Santé Publique.

Conformément à l’article L1121-4 du Code de la Santé Publique, le **Comité de Protection des Personnes** SUD-EST VI de CLERMONT-FERRAND a étudié ce projet de recherche et a émis un avis favorable à sa réalisation le 11/06/2020.

Conformément à l’article L1121-4 du Code de la Santé Publique, **l’Agence Nationale de Sécurité du Médicament et des produits de Santé** a été informée de ce projet de recherche et de l’avis favorable donné par le CPP pour sa mise en œuvre le 14/06/2020.

Si vous acceptez de participer à cette étude, merci de compléter et de signer le formulaire de consentement. Vous conserverez cette lettre d’information.

**FORMULAIRE DE CONSENTEMENT**

**pour la participation à une recherche CLINIQUE**

**VIRUS-ATTAC**

**Portage pre-operatoire de VIRUS respirAToires, et syndrome de détresse respiraToire Aiguë après chirurgie Cardiaque**

| **Catégorie de la recherche** : Recherche impliquant la personne humaine à risques et contraintes minimes | |
| --- | --- |
| Réf. CHU de Rennes : 35RC19_8869_VIRUS-ATTAC | N° ID-RCB : 2020-A00345-34 |
| **Promoteur :** CHU de Rennes  **Contact :** Direction de la Recherche et de l’Innovation  Hôpital de Pontchaillou  2 rue Henri le Guilloux  35 033 Rennes Cedex 9  Tél. : 02.99.28.25.55  **Délégué à la Protection des Données**  [dpo@chu-rennes.fr](mailto:dpo@chu-rennes.fr) | **Investigateur Coordonnateur :**  **Dr Nicolas NESSELER**  Service de Anesthésie-réanimation 3 – Réanimation CTCV  Centre cardio-pulmonaire, Hôpital Pontchaillou  2 avenue Henri Le Guilloux  35033 RENNES cedex 9  Tél. : 02.99.28.91.53 |

J’ai lu et compris la lettre d’information qui m’a été remise et que je conserve. Une information orale m’a été donnée et le médecin investigateur a répondu à toutes mes questions concernant l’étude.

Si nécessaire, je pourrai à tout moment, demander des informations complémentaires au Professeur / Docteur _………………………………………………………….…_(Tél :…………………………………).

J’ai été informé(e) de mon droit à refuser de participer à l’étude sans encourir aucun préjudice et sans que cela n’entraîne de conséquences sur la qualité des soins qui me seront prodigués.

J’ai eu le temps nécessaire pour réfléchir à mon implication dans cette étude et j’accepte librement et volontairement de participer. Cela n’engendrera aucun surcoût à ma charge.

Je peux à tout moment décider de quitter l’étude sans motiver ma décision et sans que cela n’entraîne de conséquences sur la qualité des soins qui me seront prodigués

J’ai compris que les données collectées à l’occasion de la recherche seront protégées dans le respect de la confidentialité. Elles pourront uniquement être consultées par les personnes suivantes, soumises au secret professionnel : l’équipe du médecin investigateur, les personnes mandatées par le promoteur ou les représentants des autorités de santé.

J’accepte le traitement informatisé des données à caractère personnel me concernant dans les conditions prévues par la Loi Informatique et Libertés et par le Règlement Européen 2016/679. J’ai été informé(e) de mon droit d’accès, de portabilité, de rectification, d’effacement et de limitation des données me concernant auprès du médecin investigateur.

Je certifie être affilié(e) au régime de la Sécurité Sociale, être majeur(e) et ne bénéficier d’aucun régime de protection légale (sauvegarde de justice, tutelle, curatelle).

Je suis informé(e) de la possibilité qu’une partie des prélèvements effectués à l’occasion de ce protocole de recherche soit conservée pour une utilisation ultérieure à des fins de recherche uniquement ou faire l’objet de cession à d’autres chercheurs en France ou à l’étranger. J’ai compris que les échantillons seront conservés 5 années après la dernière publication liées à VIRUS-ATTAC, puis détruits, que j’accepte ou non la réutilisation des échantillons. J’ai également été informé(e) de mon droit à m’opposer à cette conservation pour toute utilisation ultérieure.

Je suis informé(e) de la possibilité que mes données personnelles recueillies à l’occasion de ce protocole de recherche soient utilisées à des fins de recherches ultérieures. J’ai compris que chaque nouvelle recherche fera l’objet d’une information collective sur le site internet du CHU de Rennes (<https://www.chu-rennes.fr>).

J’ai également été informé(e) de mon droit à m’opposer à cette conservation pour toute utilisation ultérieure.

J’ai été informé(e) que, conformément à la réglementation sur les études cliniques, le CPP SUD EST VI de CLERMONT-FERRAND a rendu un avis favorable pour la réalisation de cette recherche le 11/06/2020 et que l’ANSM en a été informé le 14/06/2020.

Mon consentement ne décharge pas le promoteur et l’investigateur de la recherche de leurs responsabilités. Je conserve tous mes droits garantis par la loi.

| **Je soussigné(e)** Mme / M. : *(Nom Prénom)* ……………………………………………  né(e) le **└─┴─┘└─┴─┘└─┴─┴─┴─┘ atteste que le Pr / Dr :** ………………………………..  *jour mois année*  du centre de ……………………….., **m’a proposé de participer à l’étude clinique VIRUS-ATTAC.** | |
| --- | --- |
| **J’accepte librement de participer à cet essai dans les conditions précisées dans la lettre d’information :** | **OUI NON** |
| **J’accepte le partage de mes données anonymisées dans le respect de la réglementation en vigueur :** | **OUI NON** |
| **J’accepte la conservation de mes échantillons à des fins de recherches ultérieures dans le cadre de la pathologie** | **🞏 OUI NON** |
| **J’accepte l’utilisation de mes données à des fins de recherches ultérieures dans le cadre de la pathologie** | **OUI NON** |
| Le   **└─┴─┘└─┴─┘└─┴─┴─┴─┘** | **Signature du participant :** |
